# Supplementary material for: Fabrication of Orange Fluorescent Boron-Doped Graphene Quantum Dots for Al3+ Ion Detection
Source: Molecules. 2022 Oct 10;27(19):6771. doi: 10.3390/molecules27196771 (PMC9573639; doi:10.3390/molecules27196771)
Supplement: Supplementary file 1 [file molecules-27-06771-s001.zip › molecules-1913777-supplementary.pdf]

Communication

Fabrication of Orange Fluorescent Boron-Doped Graphene Quantum Dots for Al^{3+} Ion Detection

Weitao Li [1,2,*], Luoman Zhang [1], Ningjia Jiang [1], Yongqian Chen [1,*], Jie Gao [1], Jihang Zhang [1], Baoshuo Yang [1] and Jialin Liu [1,*]

[1] Textile and Garment Industry of Research Institute, Zhongyuan University of Technology, Zhengzhou 450007, China

[2] Institute of Nanochemistry and Nanobiology, School of Environmental and Chemical Engineering, Shanghai University, Shanghai 200444, China

* Correspondence: liweitao@zut.edu.cn (W.L.); chenyq@zut.edu.cn (Y.C.); liujialin60761@163.com (J.L.)

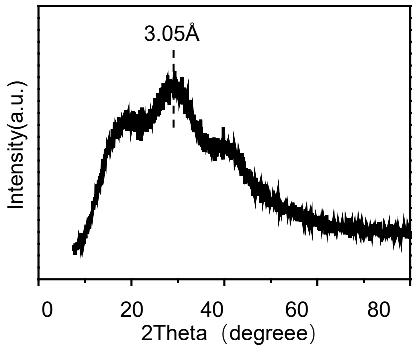

Figure S1. XRD patterns of c-GQDs.

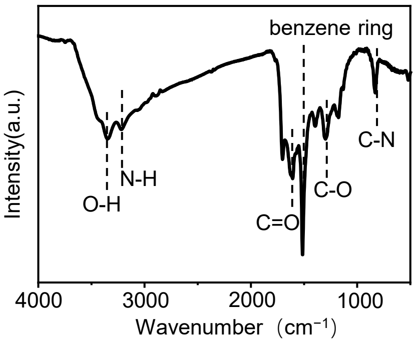

Figure S2. FT-IR spectra of c-GQDs.

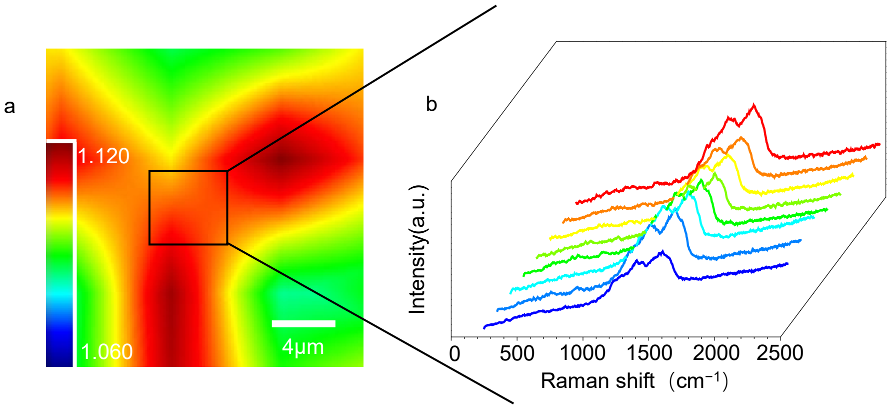

Figure S3. (**a**)High resolution microscope Raman image of c-GQDs (color indicates the ratio of peaks D to G). (**b**)Typical Raman spectra in the box region on the left.

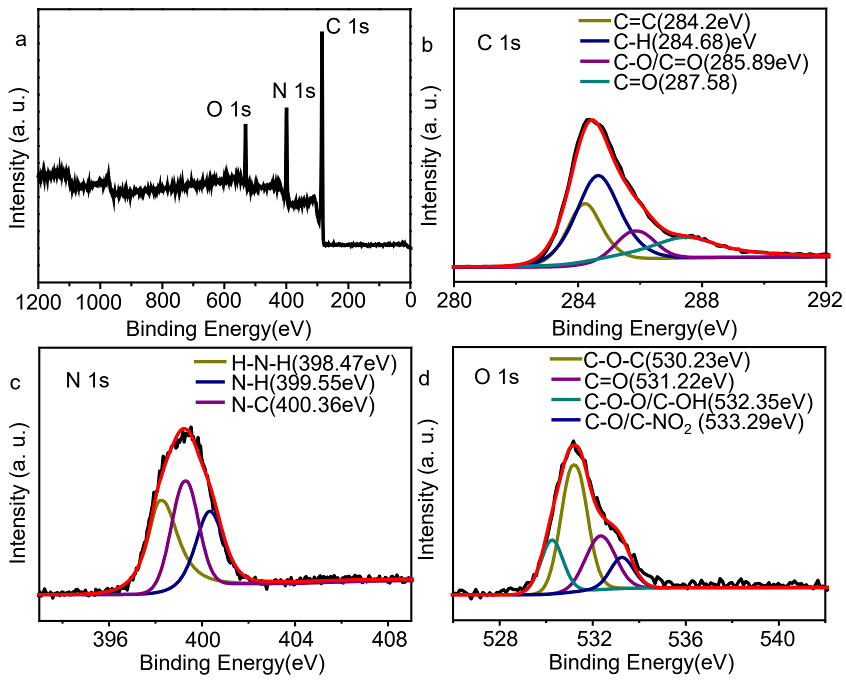

Figure S4. (**a**) Full X-ray photoelectron spectrum of c-GQDs, (**b**) C1S spectrum measured by X-ray photoelectron spectrum of c-GQDs, (**c**) N1s spectrum of c-GQDs, (**d**) O1s spectrum of c-GQDs.

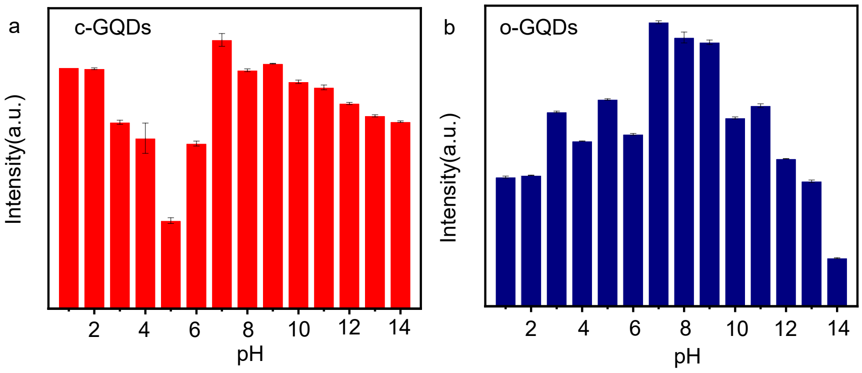

Figure S5. PH stability test of (**a**)c-GQDs, (**b**)o-GQDs.

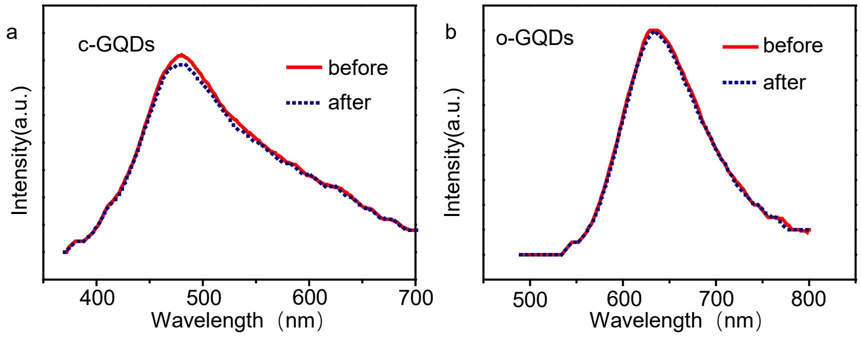

Figure S6. Dispersion stability of (**a**)c-GQDs, (**b**)o-GQDs.

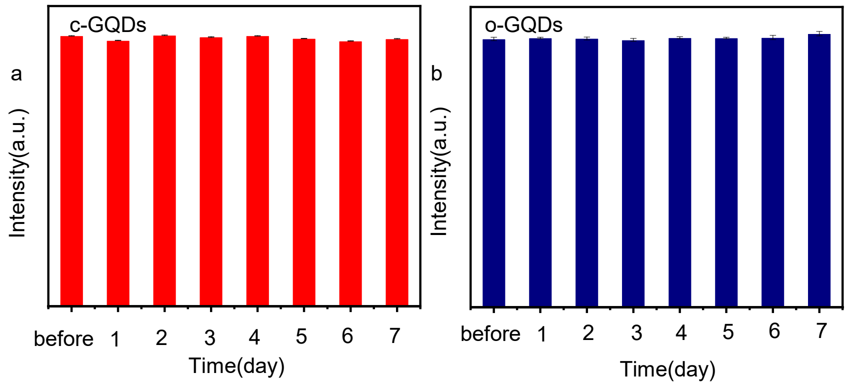

Figure S7. Temporal stability of (**a**)c-GQDs, (**b**)o-GQDs.

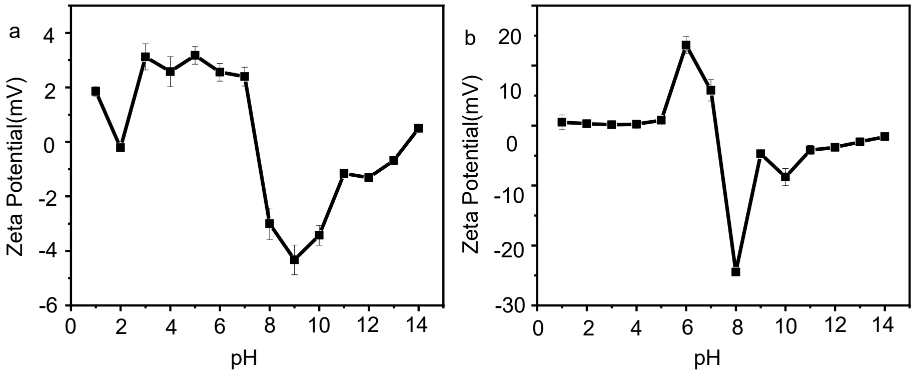

Figure S8. Zeta potential test of (**a**)c-GQDs, (**b**)o-GQDs at pH 1–14.

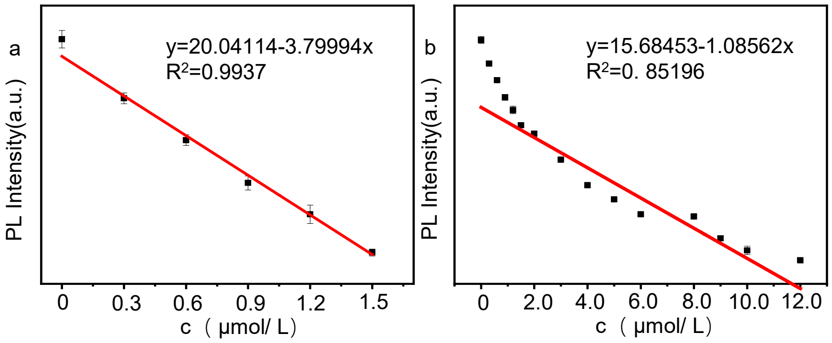

Figure S9. Linear plot between o-GQDs PL intensity and different concentrations of Al^{3+}. (**a**)Al^{3+} concentration was 0–1.5 μM; (**b**)Al^{3+} concentration was 0–12 μM.

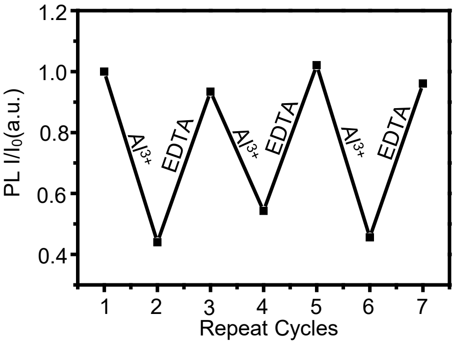

Figure S10. The reversible cycles of o-GQDs with Al^{3+} and EDTA were studied by PL intensity variation.

Table S1. XPS measures the element ratios of c-GQDs and o-GQDs in the spectrum.

Element	C 1s	N 1s	O 1s	B 1s	C 1s/O 1s	N 1s/O 1s
c-GQDs	3.5	3.58	3.63		0.96	0.98
o-GQDs	3.3	3.17	4.24	3.63	0.77	0.74
